# Large-Scale Separation of Alkaloids from *Corydalis bungeana* Turcz. by pH-Zone-Refining Counter-Current Chromatography

**DOI:** 10.3390/molecules171214968

**Published:** 2012-12-17

**Authors:** Xiao Wang, Hongjing Dong, Xikai Shu, Zhenjia Zheng, Bin Yang, Luqi Huang

**Affiliations:** 1 Shandong Analysis and Test Center, Shandong Academy of Sciences, 19 Keyuan Street, Jinan 250014, Shandong, China; 2 Institute of Chinese Material Medical, China Academy of Chinese Medical Sciences, 16 Dongzhimennei Street, Beijing 100700, China; 3 China National institute of Standardization, 4 Zhichun Road, Beijing 100088, China

**Keywords:** pH-zone-refining counter-current chromatography, large-scale separation, *Corydalis bungeana*, alkaloids

## Abstract

pH-Zone-refining counter-current chromatography (pH-zone-refining CCC) was successfully applied for the large-scale separation of alkaloids from *Corydalis bungeana*. The crude extract was separated by a two-phase solvent system composed of petroleum ether–ethyl acetate–methanol–water (5:5:2:8, v/v) where triethylamine (10 m*M*) was added to the upper organic stationary phase as a retainer and hydrochloric acid (5 m*M*) to the aqueous mobile phase as a displacer. As a result, 285 mg of protopine, 86 mg of corynoloxine, 430 mg of coryno1ine, and 115 mg of acetylcorynoline were obtained from 3.0 g of crude extract in a one-step separation. The purities of these compounds were 99.1%, 98.3%, 99.0% and 98.5%, respectively, as determined by HPLC. The chemical structures of these isolated compounds were confirmed by ESI-MS, ^1^H-NMR and ^13^C-NMR.

## 1. Introduction

*Corydalis bungeana* Turcz. (Papaveraceae) is a perennial herb distributed in many parts of the World, such as the northern and eastern parts of China and the northern part of the Korean peninsula [1]. It has been officially listed in the Chinese Pharmacopoeia for the treatment of colds and throat-swelling diseases [2]. The major bioactive constituents of *Corydalis bungeana* are alkaloids such as corynoline, acetylcorynoline, protopine, corynoloxine, *etc.* Pharmacological research showed that these alkaloids have sedative, hypnotic and bacteriostastic activity [3,4]. In addition, the bioactivities of the purified alkaloids were also studied, indicating that protopin, corynoline and acetylcorynoline can protect the liver from injury induced by CCl_4_ [5]. Therefore, it is of interest to establish an efficient method for the preparative separation and purification of alkaloids from *Corydalis bungeana* for further study of their biological activity of alkaloids.

Alkaloids present in the same plant usually exhibit similar chemical structures, which does not facilitate the preparative separation and isolation of these alkaloids. Silica gel column chromatography has been used for the separation of alkaloids from *Corydalis bungeana* [6,7,8], but this method is tedious and it usually requires multiple chromatography steps. Conventional high-speed counter-current chromatography (HSCCC) used in the elution mode was recently applied for the purification of alkaloids from *Corydalis bungeana* [9]. This technique presents many advantages such as simple operation and good reproducibility, but in this previous study, the sample size was very small (only 200 mg) and the yield was unable to meet the needs of study on the bioactive alkaloids. 

pH-Zone-refining CCC, a displacement mode of HSCCC, was introduced by Ito as a novel preparative separation technique and has been successfully applied for the large-scale purification of ionizable compounds [10,11]. As compared to conventional HSCCC, this displacement mode has many advantages such as higher sample loading capacity, higher purity of the recovered compounds and higher concentration of the collected fractions [10,12]. As far as we know, no report has been published on the use of pH-zone-refining CCC for the large-scale isolation and purification of alkaloids from *Corydalis bungeana*, so here we report an efficient method for the large-scale separation of alkaloids from *Corydalis bungeana* by pH-zone-refining CCC.

## 2. Results and Discussion

In order to obtain a large amount of purified alkaloids by pH-zone-refining CCC, the selection of a suitable two-phase solvent system which could provide suitable partition coefficient (*K*_D_) values in both acidic (*K*_acid_ << 1) and basic (*K*_base_ >> 1) conditions is very crucial [11]. According to the rule of the selection of the two-phase solvent system [10], the *K*_D_ values of two types of two-phase solvent systems including MtBE–water (1:1, v/v) were determined as previously described [11]. The results showed that both systems can provide suitable *K*_D_ values for the separation of the alkaloids. 

**Figure 1:**
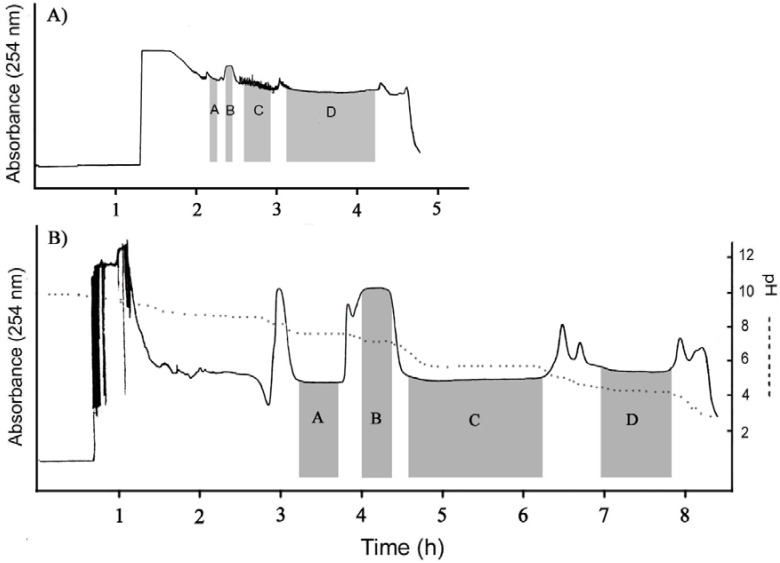
pH-Zone-refining CCC chromatograms of crude alkaloids extracts from *Corydalis bungeana*. (**A**) Two-phase solvent system: Pet–EtAc–MeOH–H_2_O (5:5:5:5, v/v), 10 m*M* TEA in the upper phase and 5 m*M* HCl in the lower phase; flow rate: 1.5 mL/min; detection wavelength: 254 nm; revolution speed: 800 rpm; sample size: 3.0 g. The retention of the final stationary phase: 38%; (**B**) Two-phase solvent system: Pet–EtAc–MeOH–H_2_O (5:5:2:8, v/v), 10 m*M* TEA in stationary phase and 5 m*M* HCl in lower phase; flow rate: 1.5 mL/min; detection wavelength: 254 nm; revolution speed: 800 rpm; sample size: 3.0 g; The retention of the final stationary phase: 68%.

After testing the two biphasic solvent systems in the CCC apparatus, the solubility of the alkaloids in the two-phase solvent system composed of MtBE–water (1:1, v/v) was too low to accomplish the large-scale separation and the amount of injected sample was no higher than 1.0 g, but the solubility of the sample in the two-phase solvent system composed of Pet–EtAc–MeOH–H_2_O (5:5:5:5, v/v) was good and the separation chromatogram presented four typical rectangular peaks as shown in Figure 1A. However, the rectangular peaks were too narrow, which means that the four alkaloids were eluted too quickly. Thus the purification process of the four alkaloids was not efficient. 

According to the rule of the selection of the solvent system, by reducing the ratio of methanol and increasing the ratio of water of the biphasic solvent system, the resolution of the target alkaloids and other impurities can be improved [11]. Thus, Pet–EtAc–MeOH–H_2_O (5:5:2:8, v/v) was selected for the efficient separation. After this two-phase solvent system was used for the CCC separation, it was found that this Pet–EtAc–MeOH–H_2_O (5:5:2:8, v/v) solvent system where triethylamine (10 m*M*) was added to the upper organic stationary phase and hydrochloric acid (5 m*M*) to the aqueous mobile phase was suitable for the large-scale separation of alkaloids. Figure 1B shows a typical chromatogram of pH-zone-refining CCC for the separation of 3.0 g of crude extract. The total separation time was about 8.5 h. Alkaloids were eluted as an irregular rectangular peak where four absorbance plateaus (plateaus A, B, C and D in Figure 1B) were observed. The measurement of pH values of the collected fractions also revealed four flat pH-zones, indicating the successful separation of four compounds. Considerable amounts of impurities were eluted in the front and back of the main peak, forming multiple peaks.

**Figure 2:**
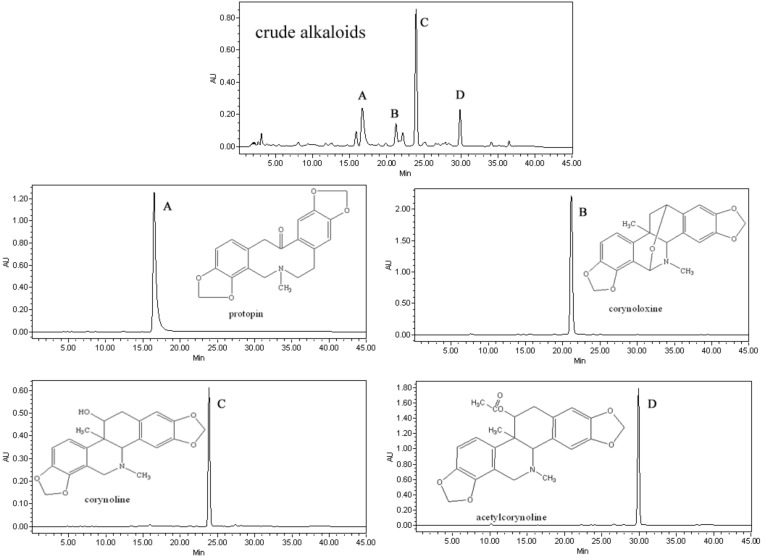
HPLC chromatograms of crude alkaloids extract from *Corydalis bungeana* and fractions of HSCCC in Figure 1B. (**A**) protopin; (**B**) corynoloxine; (**C**) coryno1ine; (**D**) acetylcoryno1ine.

Based on the HPLC analysis and elution curve of pH-zone-refining CCC chromatogram, all collected fractions were combined into different pooled fractions. The fractions obtained from CCC separation were evaporated under reduced pressure and lyophilized to dryness. As a result, 285 mg of compound A, 86 mg of compound B, 430 mg of compound C, and 115 mg of compound D were obtained in one-step separation after an injection of 3.0 g of a crude extract, with purity of 99.1%, 98.3%, 99.0% and 98.5%, respectively, as determined by HPLC and their HPLC chromatograms were shown in Figure 2. 

## 3. Experimental

### 3.1. Reagents and Materials

Petroleum ether (Pet, 60–90 °C), ethyl acetate (EtAc), methanol(MeOH), CHCl_3_, ethanol, hydrochloric acid (HCl), triethylamine (TEA), tert-butyl methyl ether (MtBE) were all of analytical grades (Jinan Luye Chemical Factory, Jinan, China). Methanol used for HPLC analysis was of chromatographic grade (Tianjin Siyou Special Reagent Factory, Tianjin, China). Reverse osmosis Milli-Q water (Millipore, USA) was used for all solutions and dilutions. *Corydalis bungeana* Turcz. was purchased from Hongjitang Drug Store (Jinan, China), and was identified by Dr. Jia Li (Shandong University of Traditional Chinese Medicine, Jinan, Shandong, China). 

### 3.2. Apparatus

CCC was carried out on a Model GS10A-2 apparatus (Beijing Emilion Science & Technology Co., Beijing, China), with a multilayer coil of 1.6 mm I.D. and 120 m in length with a total capacity of 250 mL. The *β* values of this preparative column range from 0.5 at internal to 0.8 at the external (*β = r/R*, r is the rotation radius or the distance from the coil to the holder shaft, and *R* (*R* = 8 cm) is the revolution radius or the distances between the holder axis and central axis of the centrifuge). The solvent was pumped into the column with a Model NS-1007 constant-flow pump. Continuous monitoring of the eluent was achieved with a Model 8823A-UV Monitor at 254 nm and a Model 320 pH meter (Mettler Toledo Instruments CO., Shanghai, China). A manual sample injection valve (30 mL, Tianjin High New Science Technology Company, Tianjin, China) was used to introduce the sample dissolved into the column. A portable recorder (Yokogawa Model 3057, Sichuan Instrument Factory, Chongqing, China) was used to draw the chromatogram. A Waters Millennium^32^ system including a model 996 PAD, a model 600 multi-solvent delivery system, a model 600 system controller, a model 600 pump, and a Millennium^32^ work-station (Milford, MA, USA).

### 3.3. Preparation of the Sample

Five kg of dried powder of *Corydalis bungeana* were extracted with 150 L 95% ethanol for three times (2 h each time) under reflux and then evaporated to form a syrup, which was dissolved in water (5 L) containing 1% HCl, filtered, and then partitioned with equal volumes of Pet. Ether and CHCl_3_. The CHCl_3_ extract was combined and evaporated to dryness by a rotary vaporization at 50 °C yielding 27.1 g of crude alkaloids.

### 3.4. Separation Procedure 

The two-phase solvent was equilibrated in a separatory funnel, and separated before use. The lower phase (mobile phase) was acidified with HCl to give a 5 mM solution. The upper phase (stationary phase) was rendered basic by addition of TEA to give a 10 mM solution. The separation was initiated by filling the entire column with the stationary phase, and then loading the sample dissolved in a mixture of stationary and aqueous phases (in the ratio of 3:1). The mobile phase was then pumped into the column at 1.5 mL/min while the column was rotated at 800 rpm in the head to tail elution mode. The absorbance of the eluent was continuously monitored at 254 nm and 5 mL fractions were collected. The pH of each eluted fraction was measured with a pH meter.

### 3.5. HPLC Analysis of pH-Zone-refining CCC Peak Fractions 

HPLC analysis of the crude alkaloids and each purified fraction were performed with a Welch Materials C_18_ (250 × 4.6 mm, i.d., 5 μm) column at room temperature. The mobile phase was a solution of methanol-aqueous containing 0.1% TEA as follows: 0–30 min, 60% to 90% methanol, 30–35 min, 90% to 100% methanol, 35–40 min, 100% to 60% methanol, 40–45 min, 60% methanol. The eluent was monitored by a PAD at 280 nm, and the flow-rate was 1.0 mL/min. The identification of each purified compound was carried out by electrospray ionization-mass spectrometry (ESI-MS) on an Agilent 1100LC/MSD (Agilent, Palo Alto, CA, USA), and ^1^H-NMR and ^13^C-NMR spectra on a Varian INOVA 600 spectrometer (Varian, Palo Alto, CA, USA) with tetramethysilane (TMS) as internal standard.

### 3.6. Identification of Compounds

Peak A: ESI-MS: *m/z* 354 [M+H]^+^. ^1^H-NMR (600 MHz, CDCl_3_) *δ* (ppm): 6.90 (1H, s, H-1), 6.69 (1H, d, *J* = 7.8 Hz, H-12), 6.66 (1H, d, *J* = 7.8 Hz, H-11), 6.64 (1H, s, H-4), 5.95 (2H, s, O-CH_2_-O), 5.92 (2H, s, O-CH_2_-O), 3.80 (2H, br, H-13), 3.59 (2H, br, H-8), 2.91 (2H, br, H-5), 2.56 (2H, br, H-6), 1.93 (3H, S, NCH_3_). ^13^C-NMR (150 MHz, CDCl_3_) *δ* (ppm): 194.7 (C-14), 148.0 (C-3), 146.3 (C-2), 146.0 (C-9), 145.9 (C-10), 136.0 (C-4a), 132.7 (C-14a), 128.9 (C-12a), 125.0 (C-12), 117.8 (C-8a), 110.5 (C-4), 108.1 (C-1), 106.8 (C-11), 101.2 (O-CH_2_-O), 100.9 (O-CH_2_-O), 57.8 (C-6), 50.9 (C-8), 46.4 (C-13), 41.5 (NCH_3_), 31.7 (C-5). The results were the same to those in ref. [13], the compound was identified as protopine.

Peak B: ESI-MS: *m/z* 366 [M+H]^+^. ^1^H-NMR(600 MHz, DMSO-d_6_) *δ* (ppm): 6.75 (1H, s, H-1), 6.78 (1H, s, H-4), 6.85 (1H, d, *J* = 7.8 Hz, H-9), 6.90 (1H, d, *J* = 7.8 Hz, H-10), 6.07, 6.04(each 1H, d, *J* = 1.2 Hz, 2-O-CH_2_-O), 5.96(2H, d, *J* = 1.2 Hz, 7-O-CH_2_-O), 1.98(3H, s, 5-NCH_3_), 5.07(1H, s, H-6), 3.49(1H, d, *J* = 1.8 Hz, H-11), 2.89 (2H, d, *J* = 2.4 Hz, H-12), 2.83(1H, d, *J* = 1.8 Hz, H-14). 1.17(3H, s, H-13CH_3_). ^13^C-NMR (150 MHz) *δ* (ppm): 146.8 (C-8), 146.5 (C-3), 145.6 (C-2), 140.9 (C-7), 134.7 (C-10a), 131.2 (C-4a), 124.9 (C-1a), 119.0 (C-6a), 115.0 (C-10), 109.8 (C-9), 109.3 (C-4), 107.0 (C-1), 101.7 (2-O-CH_2_-O), 101.0 (7-O-CH_2_-O), 77.3 (C-6), 71.8 (C-11), 63.2 (C-14), 39.4 (5-NCH_3_), 36.6 (C-13), 32.3 (C-12), 15.8 (15-CH_3_). The results were same to those in ref. [8], and it was identified as corynoloxine.

Peak C: ESI-MS: *m/z* 368 [M+H]^+^. ^1^H-NMR(600 MHz, CDCl_3_) *δ* (ppm): 7.09 (1H, s), 6.87 (1H, d, *J* = 8.4 Hz),6.79 (1H, d, *J* = 7.8 Hz), 6.63(1H, s), 5.99 (1H, s), 5.94 (2H, s), 4.23 (1H, d, *J* = 15.6 Hz), 4.13 (1H, m), 3.34 (1H, d, *J* = 18.0 Hz), 3.04 (1H, dd, *J* = 4.2, 18.0 Hz), 2.64 (3H, s), 1.16 (3H, s). ^13^C-NMR (150 MHz) *δ* (ppm): 22.7 (C14-CH_3_), 35.4 (C-12), 40.5 (C-14), 41.8 (N-CH_3_), 52.9 (C-6), 69.1 (C-13), 75.3 (C-11), 101.3 (-OCH_2_-O-), 101.7 (-OCH_2_-O-), 108.5 (C-9), 109.4 (C-1), 118.9 (C-10), 113.2 (C-4), 118.9 (C-10), 128.3 (C-4a), 133.8 (C-10a), 142.9 (C-7), 145.6 (C-8), 149.0 (C-3). The results were same to those in ref. [9], and it was identified as coryno1ine.

Peak D: ESI-MS: *m/z* 410 [M+H]^+^. ^1^H-NMR (600 MHz, CDCl_3_) *δ* (ppm): 7.29 (1H, s), 6.89 (1H, d, *J* = 8.4 Hz), 6.85 (1H, d, *J* = 8.4 Hz), 6.69 (1H, s), 6.06 (1H, s), 6.00 (1H, s), 5.99 (2H, s), 4.74 (1H, d, *J* = 16.2 Hz), 4.40 (1H, d, *J* = 16.2 Hz), 3.21 (1H, dd, *J* = 4.2, 18.0 Hz), 2.64 (3H, s), 1.73 (3H, s), 1.73 (3H, s), 1.16 (3H, s). ^13^C-NMR (150 MHz) *δ* (ppm): 21.0 (C14-CH_3_), 29.8 (-CH_3_), 31.2 (C-12), 35.7 (C-14), 39.0 (N-CH_3_), 49.8 (C-6), 64.3 (C-13), 71.8 (C-11), 101.7 (OCH_2_-O-), 102.2 (-OCH_2_-O-), 108.6 (C-9), 109.4 (C-1), 118.3 (C-6a), 110.5 (C-4), 118.9 (C-10), 126.6 (C-4a), 130.2 (C-10a), 145.3 (C-7), 146.2 (C-8), 148.1 (C-2), 149.03 (C-3), 169.2 (C = O). The results were same to those in ref. [14], and it was identified as acetylcoryno1ine.

## 4. Conclusions 

In present study, pH-zone-refining CCC was successfully applied for the large-scale separation of protopine, corynoloxine, coryno1ine and acetylcoryno1ine from *Corydalis bungeana* in a one-step separation. The overall results in our work clearly demonstrated that the selection of a suitable two-phase solvent in which the solubility of the sample should be high is very crucial in the large-scale separation of pH-zone refining CCC separation. 

## References

[1] Chen X., Nigel C.V., Peter J.H., Monique S.S. (2004). Flavonoid glycosides and isoquinolinone alkaloids from *Corydalis bungeana*. Phytochemistry.

[2] China Pharmacopoeia Committee (2010). Pharmacopoeia of the People’s Republic of China, First Division of the 2010 Edition.

[3] Liu X.J., Zhang H.L., Tan Z.C., Han K.L., Sun L.X. (2007). Mircocalorimetric study on the bacteriostatic activity of isoquinoline alkaloids. J. Therm. Anal. Calorim..

[4] Yang J.G., Yuan H.N., Che J., Zhang Q. (1990). Preliminary observations of the sedative and hypnotic effects from alkaloids in *Corydalis bungeana*. J. Clin. Pharmacol..

[5] Wei H.L., Liu G.T. (1997). Protective action of corynoline, Acetylcorynoline and protopine against experimental liver injury in mice. Yao Xue Xue Bao.

[6] Zheng J.F., Qin M.J., Zheng Y., Chen L.C. (2007). Alkaloids from *Corydalis bungeana*. J. China Pharm. Univ..

[7] Huang G., Li F.M. (2003). The separation and structure identification of alkaloids in *Corydalis bungeana* Turcz. Chin. Tradit. Herbal Drugs.

[8] Huang G., Yang H.J., Li F.M. (2003). Preparation and simultaneous determination of corynoline and acetylcorynoline in the herb of *Corydalis bungeana*. China J. Chin. Mater. Medica.

[9] Niu L.L., Xie Z.S., Cai T.X., Wu P., Xue P., Chen X.L., Wu Z.Y., Ito Y., Li F.M., Yang F.Q. (2011). Preparative isolation of alkaloids from *Corydalis bungeana *Turcz. by high-speed counter-current chromatography using stepwise elution**. J. Sep. Sci..

[10] Ito Y. (2005). Golden rules and pitfalls in selecting optimum conditions for high-speed counter-current chromatography. J. Chromatogr. A.

[11] Fang L., Liu Y.Q., Yang B., Wang X., Huang L.Q. (2011). Separation of alkaloids from herbs using high-speed counter-current chromatography. J. Sep. Sci..

[12] Hu R., Pan Y. (2012). Recent trends in counter-current chromatography. TRAC-Trend. Anal. Chem..

[13] Yu X.H., Wang Z.T., Yu G.D., Ruan B.F., Li J. (2002). Alkaloids from Rhizoma corydalis. J. China Pharm. Univ..

[14] Zeng W.G., Liang W.Z., He C.H., Zhang Q.T., Tu G.S. (1988). An alkaloid from *Corydalis bungeana*. Phytochemistry.

